# The Role of IL-6 in Fibrotic Diseases: Molecular and Cellular Mechanisms

**DOI:** 10.7150/ijbs.75876

**Published:** 2022-08-29

**Authors:** Yanxia Li, Jing Zhao, Yuan Yin, Ke Li, Chenchen Zhang, Yajuan Zheng

**Affiliations:** Department of Ophthalmology, The Second Hospital of Jilin University, Jilin University, Changchun, China

**Keywords:** Interleukin 6, JAK, STAT3, fibrosis

## Abstract

Fibrosis is a detrimental outcome of most chronic inflammatory disorders and is defined by the buildup of excess extracellular matrix (ECM) components, which eventually leads to organ failure and death. Interleukin 6 (IL-6) is promptly produced by immune cells in response to tissue injuries and has a wide range of effects on cellular processes such as acute responses, hematopoiesis, and immune reactions. Furthermore, high levels of IL-6 have been found in a variety of chronic inflammatory disorders characterized by fibrosis, and this factor plays a significant role in fibrosis in various organs via Janus kinase/signal transducer and activator of transcription 3 (JAK/STAT3) activation. Here, we review what is known about the role of IL-6 in fibrosis and why targeting IL-6 for fibrotic disease treatment makes sense.

## Introduction

Fibrosis or scarring, which is defined as the excessive accumulation of fibrous connective tissue, is a common pathological condition resulting from a dysregulated tissue repair response, most notably during chronic inflammatory disorders 1. Myofibroblasts, the most important cell type initiating the wound healing response when tissues are injured, are characterized by the expression of alpha-smooth muscle actin (α-SMA); these cells have high contractility, secrete inflammatory factors, and deposit extracellular matrix (ECM) components, such as fibronectin and collagen 2. Fibrosis affects nearly all tissues and organs in the body. Although fibrogenesis is a major cause of adverse prognosis in most tissue repair processes or chronic inflammatory disorders, few therapeutic options are available to prevent or reverse this process 3. As a result, more efficient antifibrotic therapies to reduce mortality and morbidity and developing a greater understanding of the molecular mechanisms related to fibrosis deserve further attention.

Interleukin-6 (IL-6) is a multifunctional cytokine that is involved in a variety of biological processes, including inflammation, immunological response, and hematopoiesis 4. Most importantly, IL-6 is crucial for the inflammatory phase and the switch to a reparative environment during the resolution of wound healing 5. However, the consequences of repair will eventually lead to fibrosis if the switch to the proliferative phase is uncontrolled. IL-6 is a key regulator of inflammation and repair, and its role in scar formation has been demonstrated in previous studies.

In this review, we summarized recent research investigating the role of IL-6 in fibrosis and discuss how to block IL-6 signaling to reduce fibrosis.

## The IL-6/IL-6R system and signal transduction

Because some excellent reviews on this topic have examined IL-6 signaling in great detail, the general mechanisms of IL-6 signaling are only briefly introduced here. Many cell types, including monocytes, macrophages, fibroblasts, keratinocytes, astrocytes, and endothelial cells, can secrete IL-6, which is a four-helical cytokine of 184 amino acids 6. The IL-6 receptor (IL-6R) and glycoprotein 130 (gp130) are responsible for the biological effects of IL-6^7^. Although gp130 is expressed universally, IL-6R is expressed primarily by hepatocytes, leukocytes, and megakaryocytes 6. There are two types of IL-6R, membrane-bound IL-6R (mIL-6R) and soluble IL-6R (sIL-6R), which mediate the classic signaling pathway and trans-signaling pathway, respectively 7-9. IL-6 binding to mIL-6R induces gp130 homodimerization, which leads to the formation of a high-affinity functional receptor complex of IL-6-mIL-6R-gp130 in the classic signaling pathway. sIL-6R can also bind to IL-6, and the sIL-6R-IL-6-gp130 complex can result in intracellular signaling similar to that of mIL-6R. Importantly, the presence of sIL-6R can stimulate downstream signals in cells that do not express mIL-6R; this unique receptor signaling mechanism has been called the IL-6 trans-signaling pathway 10.

**Fig 1:**
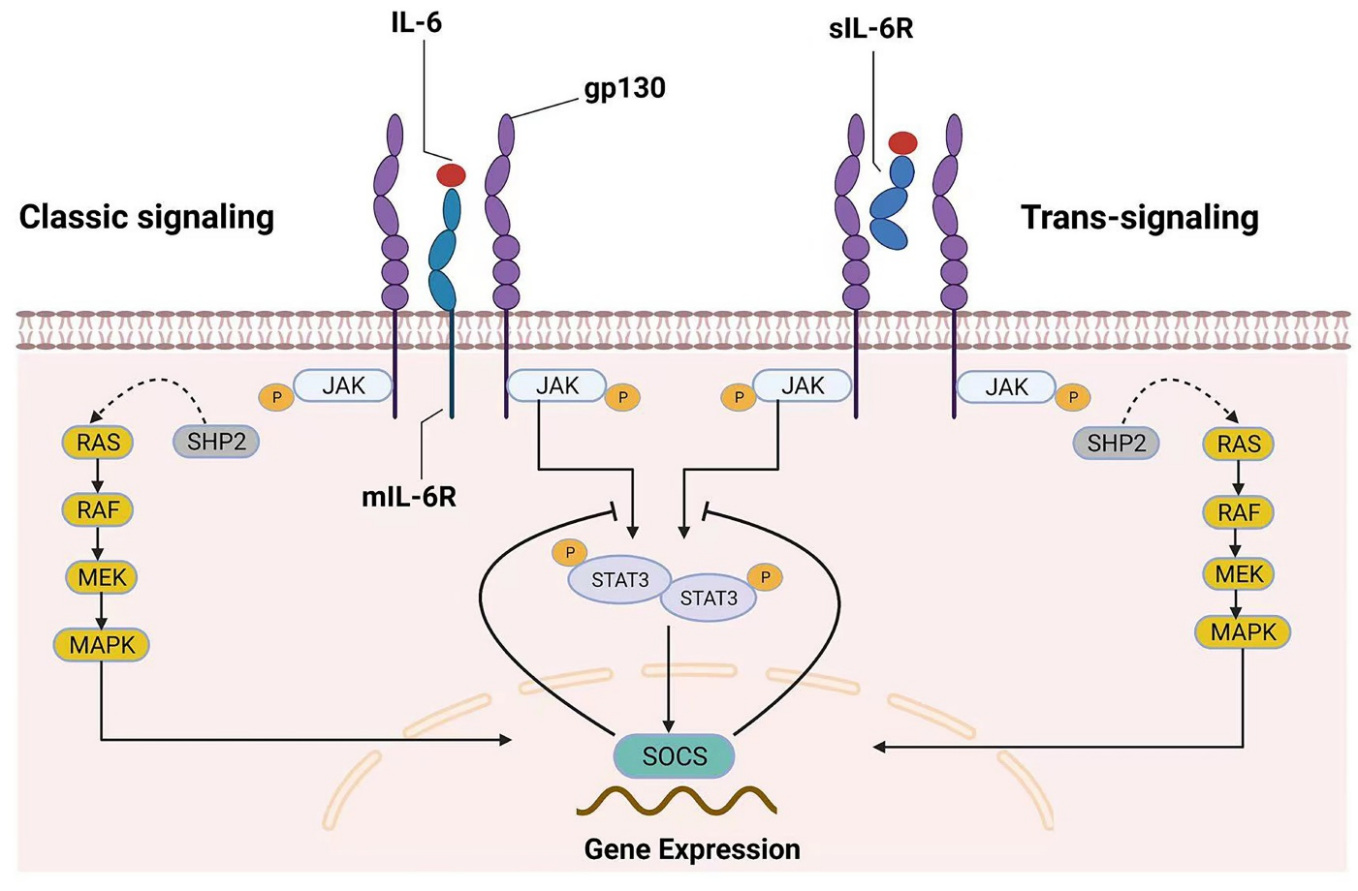
**Two different modes of IL‑6 signaling.** IL-6 can bind to both mIL‑6R (classic signaling) and sIL-6R (trans-signaling). After homodimerization of the signal-transducing receptor subunit gp130 on the plasma membrane, the two signaling pathways converge, triggering the intracellular JAK/STAT and MAPK signaling cascades. SOCS is the product of JAK/STAT3 signaling pathway activation and is also a protein associated with the negative-feedback reaction that leads to the inhibition of this signaling pathway.

Generally, the pathway that involves tyrosine kinases in the Janus kinase (JAK) family and transcription factors in the signal transducer and activator of transcription (STAT) family (the JAK/STAT pathway) and the mitogen-activated protein kinase (MAPK) signaling cascades are activated once the IL-6 receptor complex is engaged 11. In the JAK/STAT pathway, STAT proteins are recruited and phosphorylated by JAKs in response to IL-6 stimulation. Subsequently, activated STAT proteins form dimers and translocate to the nucleus to trigger the transcription of target genes 7, such as those encoding regulators of cellular proliferation (such as cyclin D1, cyclin B1 and c-myc) and survival (such as survivin, BCL-2 and BCL-xL), as well as angiogenic factors (such as VEGF and HIF1α) and cytokines (such as IL-10, IL-11 and IL-17) 10, 12. Interestingly, suppressor of cytokine signaling (SOCS), one of the target genes of the JAK/STAT pathway, can inhibit JAK activity and thus negatively regulate this pathway, suggesting that this signaling pathway has an autoregulatory mechanism 7, 13. On the other hand, SHP-2 is recruited to the phosphorylated Tyr759 residue of gp130 and is then phosphorylated by JAKs 7, 11, 14. Subsequently, SHP-2 interacts with the growth-factor-receptor-bound protein 2/Son of Sevenless (Grb2-SOS) complex 7, 11, 15, which is a GDT/GTP exchanger for Ras. Finally, the Raf-Ras-MAPK cascade is activated after IL-6 stimulation. IL-6 binding to mIL-6R/sIL-6R activates the downstream signaling pathway, as shown in Figure 1.

## The role of IL-6 in wound healing

An overly active wound healing response frequently results in tissue fibrosis, and wound repair has been divided into three primary stages 16. First, the innate immune system is activated and inflammatory cytokines are released in the early inflammatory phase; the proliferative phase is initiated by an influx of fibroblasts and the transdifferentiation of fibroblasts to myofibroblasts, resulting in wound contraction and ECM deposition; with wound closure, type III collagen degrades and type I collagen synthesis increases, the tissue gains strength and flexibility, and the remodeling phase begins and can last for months to years 5, 16. To engage in wound repair, IL-6 is expressed during two periods in wound sites. It is rapidly upregulated following injury, and robust levels peak at approximately 12 hours, but by 24 hours, IL-6 levels return to near baseline levels 17, 18. After approximately 48 hours, the second phase of IL-6 expression in the wound commences, and cytokine levels progressively reach a stable state at 3-7 days postinjury, after which the concentration returns to normal levels in the remodeling phase 17, 18.

Inflammation can be collectively activated by the clotting response, necrotic debris, and invading bacteria after injury. Neutrophils, monocytes, and other innate immune cells initially accumulate at the wound site to clear cell debris and infectious organisms and secrete proinflammatory cytokines and growth factors to aid in the tissue healing response. The wound inflammatory response, on the other hand, can become dysregulated or chronic, leading to pathological fibrosis or scarring, which can disrupt normal tissue architecture and function 19. As a significant modulator of inflammatory and reparative processes, IL-6 plays a critical role in wound healing. Compared to wild-type mice, transgenic IL-6-deficient mice exhibited impaired wound healing as well as a reduction in leukocyte infiltration, angiogenesis, re-epithelialization, and collagen accumulation at damage sites 20, 21. Furthermore, in wild-type mice, treatment with a neutralizing anti-IL-6 monoclonal antibody significantly delayed wound closure 21. Specifically, IL-6 is expressed in neutrophils, macrophages and fibroblasts 22 and participates in wound healing by regulating the activity of these cells. sIL-6R can be shed from the surface of neutrophils into the wound 23. IL-6/sIL-6R complexes further trigger the recruitment of macrophages into wound tissues by increasing the expression of certain chemokines, such as monocyte chemoattractant protein 1 (MCP-1) 24, 25. IL-6 then promotes the polarization of proinflammatory M1 macrophages to reparative M2 macrophages, as well as their proliferation 6, 26. Importantly, a switch in macrophages from the M1 to the M2 phenotype is a significant step in the transition of wound healing from the inflammatory phase to the proliferative phase. Furthermore, IL-6 can drive fibroblast migration to injury sites and regulate fibroblast differentiation to myofibroblasts through the JAK/ERK pathway or via paracrine production of transforming growth factor β (TGF-β) at wound sites 5.

## IL-6-induced cellular processes associated with fibrosis

The roles of various cellular mechanisms in the pathogenesis of fibrosis, as well as their relationship with IL-6, are discussed below.

The process by which fibroblasts transform into myofibroblasts is known as fibroblast-to-mesenchymal transition (FMT), which is characterized by the expression of α-SMA and excessive deposition of ECM. Accumulating evidence suggests that FMT could be induced by IL-6 in fibroblasts derived from different tissues. In cultured cardiac fibroblasts exposed to exogenous recombinant hypoxia-induced mitogenic factor, the increased production of IL-6 induced cell proliferation, migration, and myofibroblast differentiation through the MAPK and Ca^2+^/calmodulin-dependent protein kinase II (CaMKII)-STAT3 pathways 27. Hepatic stellate cells (HSCs) are mesenchymal cells that retain features of fibroblasts, which are the major cellular source of myofibroblasts and the major driver of liver fibrosis 28. HSCs treated with IL-6 upregulate the expression of α-SMA and collagen and induce the phenotypic transition of quiescent HSCs toward myofibroblast-like cells 29. Wheeler 30 also suggested that IL-6 trans-signaling might be involved in regulating fibrosis genes in lung mesenchymal cells, causing increased invasion and fibrotic differentiation. These findings indicate that IL-6 might drive fibrosis in various human fibrotic diseases.

Epithelial-mesenchymal transition (EMT) is another biological process induced by IL-6 in fibrotic diseases, in which epithelial cells lose their epithelial phenotypes and gain mesenchymal phenotypes with increased migration and invasion capacities 31. EMT plays a crucial role in embryonic development and wound healing and contributes pathologically to fibrosis and cancer progression. Exogenous addition of IL-6 to ovarian cancer cells activated STAT3 and mesenchymal cell marker expression and enhanced cell motility 32. Furthermore, neutralizing IL-6 signaling was sufficient to reverse the EMT characteristics of human proximal tubular epithelial cells induced by PM2.5 33. In peritoneal fibrosis, IL-6 also stimulates EMT 34.

Apoptosis is a type of programmed cell death that maintains the homeostasis of many adult tissues by regulating cell numbers. Generally, myofibroblasts are cleared from wound sites through apoptosis as repair is completed under normal conditions. Thus, myofibroblast apoptosis is a critical process in the resolution of fibrosis. However, myofibroblasts from pathological fibrotic tissues are usually resistant to apoptosis, leading to persistent accumulation of ECM and contraction of the tissue. IL-6, which is a growth or survival factor, has been shown to play a critical role in apoptosis resistance by inducing antiapoptotic proteins, such as survivin, Bcl-xL and Mcl-1 35. In non-small cell lung cancer (NSCLC), the addition of IL-6 to NSCLC cells could improve their resistance to apoptosis 35. IL-6 can also have different effects on apoptosis in different types of cells, tissues and organs. Moodley et al. 36 demonstrated that in fibroblasts without lung disease, IL-6 could enhance FasL-induced apoptosis via STAT3. Treatment of idiopathic pulmonary fibrosis (IPF) fibroblasts with IL-6, on the other hand, conferred resistance to FasL-induced apoptosis. It was thought that the contrasting effects of IL-6 were linked to differential activation of STAT3 and ERK.

Autophagy is a process that maintains cell survival by delivering dysfunctional cellular cytoplasmic components to lysosomes for degradation 37. Recently, evidence has indicated that autophagy dysregulation is related to diverse types of pathologic conditions, including fibrotic processes. Autophagy defects in human microvascular endothelial cells (HMVECs) induce EMT 38. Furthermore, the IL-6 level was significantly higher due to autophagy dysregulation. In vitro experiments also showed that endothelial-specific autophagy related 5 (atg5)-knockout mice developed kidney and heart fibrosis with an increase in serum IL-6 levels 38. In Takayasu's arteritis, IL-6 significantly promotes autophagy and fibrosis via the JAK1 pathway 39.

## The role of IL-6 in fibrosis in various tissues

**Fig 2:**
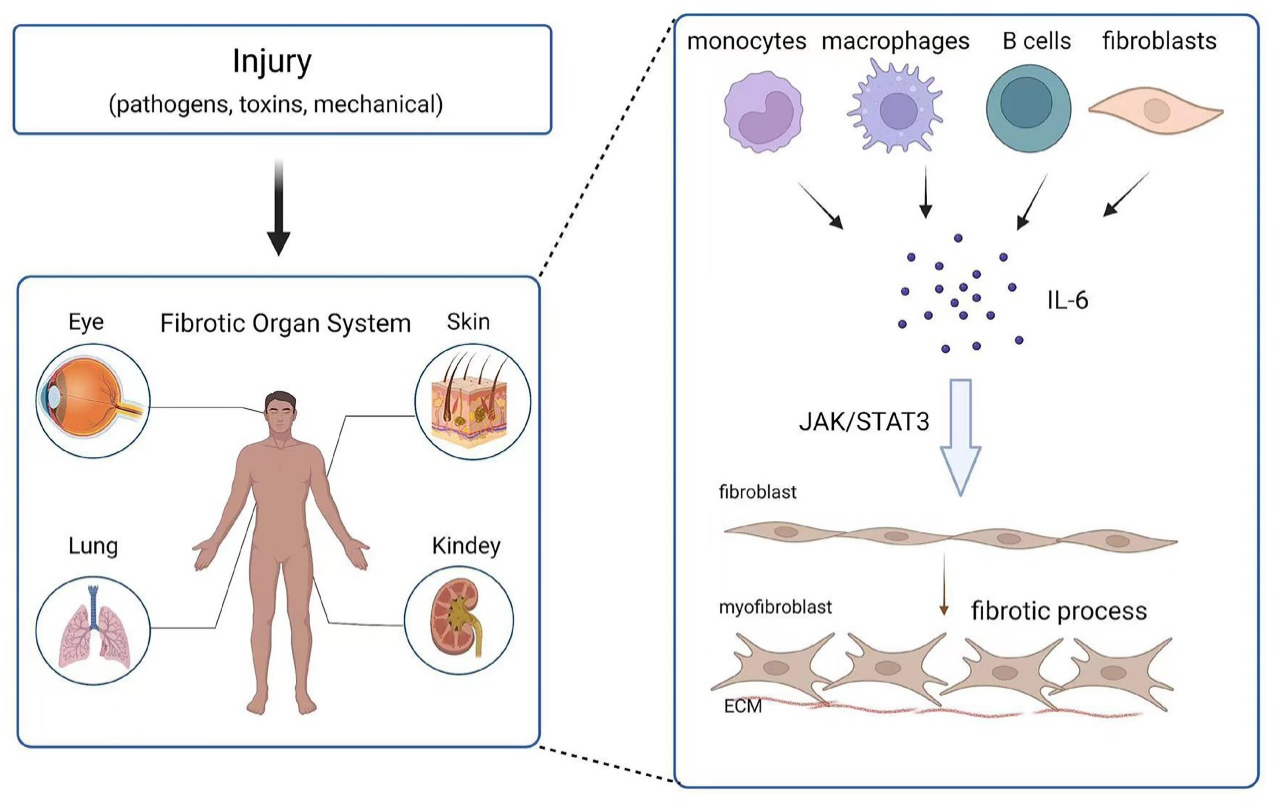
** Effect and mechanism of IL-6 on organ fibrosis.** Chronic inflammation is caused by injury (pathogens, toxins, mechanical injury), and monocytes, macrophages, B cells and fibroblasts release IL-6 at the wound site. IL-6 promotes the transformation of fibroblasts into myofibroblasts through the JAK/STAT3 signaling pathway.

Fibrosis can affect any system and organ in the body. The role of IL-6 in fibrosis in different tissues and organs has been reported (Figure 2). In the following section, the contribution of IL-6 to the development of fibrosis in various tissues is detailed.

### IL-6 and lung fibrosis

Pulmonary fibrosis occurs in a wide range of clinical contexts, is a leading cause of morbidity and mortality, and is a major unmet medical need 40. The morphological characteristics of pulmonary fibrosis are diffuse inflammatory infiltration and alveolar septal thickening with collagen accumulation and myofibroblast proliferation 41. IPF is a chronic and progressive interstitial pneumonia of unknown cause 42 and is a representative disease of pulmonary fibrosis. IL-6 was shown to be upregulated in IPF patients 43 and animal models of pulmonary fibrosis 44; furthermore, JAK2 and STAT3 were increased and activated in the hyperplastic alveolar cells of IPF patients 42, which suggested that IL-6/JAK/STAT3 plays a role in lung fibrosis. In animal studies, Saito et al. 41 found that IL-6-deficient mice had relatively attenuated fibrosis following bleomycin administration, which is a well-characterized animal model of pulmonary fibrosis, in comparison to the wild-type controls. Consistently, IL-6 overexpression could increase the fibrotic response to bleomycin, such as collagen production, lung elasticity and fibrotic scores 45. In terms of cellular mechanisms, alveolar epithelial cell damage is a key stage in the pathogenesis of lung fibrosis, as it can lead to dysregulated activation and establish a profibrotic microenvironment 46, 47. Type II alveolar epithelial cell injury can be reduced by inhibiting the STAT3 pathway 47, indicating that IL-6 may participate in pulmonary fibrosis through alveolar epithelial cells. Lung fibroblasts are another important cell type involved in the pathogenesis of pulmonary fibrosis. Pechkovsky showed that collagen I secretion in IPF lung fibroblasts was regulated by STAT3 and enhanced collagen I expression, which might be responsible for their fibrogenic phenotype 48. These results suggest that IL-6/JAK/STAT3 may be a potential therapeutic target for treating pulmonary fibrosis.

The global outbreak of coronavirus disease 2019 (COVID-19) has seriously endangered healthcare systems worldwide. Fibrotic manifestations resulting from lung injury 49, 50 are commonly associated with severe injury, and some of the molecular markers, such as TGF-β and IL-6, are increased in the peripheral blood of severe COVID-19 patients 51. These findings suggest that pulmonary fibrosis plays a role in the progression of COVID-19. Indeed, it is possible that COVID-19-induced lung injury in patients with severe disease might trigger an abnormal wound healing response that leads to the deposition of fibrotic tissue and eventually develops into pulmonary fibrosis 52. Several studies suggest that an elevated level of IL-6 correlates with a severe response to COVID-19 53, 54; that is, IL-6 may be a predictor for COVID-19 patients who develop pulmonary fibrosis. In fact, tocilizumab, a humanized anti-IL-6R antibody that binds to both mIL-6R and sIL-6R for total IL-6 signal transduction suppression, has been used to treat critically ill patients with COVID-19 55. Pulmonary computed tomography imaging in 23 patients with severe COVID-19 who were treated with tocilizumab showed improvements without adverse events according to a retrospective study from China 56. Another observational study reported that patients with severe COVID-19 who received an intravenous tocilizumab dose of 8 mg/kg (maximum 800 mg) had survival rates comparable to those in the nonsevere group 57. These findings point to the therapeutic potential of tocilizumab in preventing long-term fibrotic repercussions by reducing lung damage, which warrants further investigation.

### IL-6 and renal fibrosis

Because of the high morbidity and mortality associated with chronic kidney disease (CKD), CKD is a serious public health concern 58. Renal fibrosis, which represents the common final pathway of CKD, is characterized by fibroblast activation and ECM deposition and results in renal parenchyma injuries and renal function loss 59. Recently, a large body of experimental evidence suggested that IL-6 plays a role in renal fibrosis, although this finding remains controversial. IL-6 was significantly increased in an animal model of unilateral ureteral obstruction (UUO)-induced kidney fibrosis; nevertheless, IL-6-knockout mice exhibited comparable expression levels of ECM proteins in the kidneys following obstructive damage compared to wild-type animals 60. Contrary to these results, IL-6-deficient mice had significantly attenuated angiotensin II-induced collagen staining and total collagen levels 61. In another study, Chen et al. 59 used Fc-gp130, which binds to sIL-6R, to block IL-6 trans-signaling in the UUO mouse model of renal fibrosis and found significantly reduced renal fibrosis and anti-inflammatory effects, as evidenced by decreased ECM protein synthesis and immune cell infiltration. A plausible explanation for the contradictory findings is that the classic signaling pathway is anti-inflammatory and the trans-signaling pathway is proinflammatory, and the effects of the two pathways may counteract each other in IL-6-knockout mice following obstructive injury; although renal fibrosis might be reduced due to the absence of proinflammatory trans-signaling, injured renal tissue also lacks the anti-inflammatory effects of classic IL-6 signaling, and fibrosis can probably be exacerbated by an increase in other inflammatory processes 59.

In end-stage renal disease, peritoneal dialysis, which is a common therapeutic method, has drawn increasing attention 62. However, long-term use of the patient's peritoneal membrane as a dialyzer filter is unphysiological and results in chronic inflammation, which eventually develops into peritoneal fibrosis 63. In patients with peritoneal dialysis, it was reported that elevated circulating levels of IL-6 indicated a poor outcome 64. In addition, IL-6 has been shown to promote EMT in human peritoneal mesothelial cells, which are involved in fibrotic processes. For example, Xiao et al. 34 found that IL-6 overexpression may cause morphological changes in fibroblast-like cells among human peritoneal mesothelial cells through the JAK2/STAT3 signaling pathway. Another study illustrated the mechanism of IL-6-induced EMT from an epigenetic standpoint. The authors found that exposing human peritoneal mesothelial cells to IL-6 increased the expression of histone deacetylase 6, which not only regulated IL-6 downstream of JAK2/STAT3 signaling but also activated TGF-β/Smad3 signaling, resulting in a change in the phenotype and increased cell migration 65.

### IL-6 and dermal fibrosis

Hypertrophic scars and keloids represent an aberrant response of the skin to the wound healing process 66. One main histological characteristic of hypertrophic scars is the hypercellularity caused by persistent inflammation during early wound healing and after wound closure 67. In patients with hypertrophic scars, Tyr705 STAT3 phosphorylation is elevated 68. Furthermore, IL-6/sIL-6R administration to fibroblasts derived from hypertrophic scars and the nonburned area of the same patient both resulted in the production of ECM and the upregulation of the cellular proliferation markers cyclin D1, Bcl-Xl and c-Myc 68. However, hypertrophic scar fibroblasts have enhanced sensitivity to IL-6 trans-signaling. These results implicated the IL-6 trans-signaling-STAT3 pathway in hypertrophic scar pathogenesis. Unlike hypertrophic scars, keloids can emerge several years later and grow in a tumor-like manner beyond the borders of the original lesion 66, 69. In fibroblasts isolated from patients with keloids, increased gene expression and protein production of IL-6 was identified 70, 71. Furthermore, inhibiting IL-6 with the corresponding antibodies in keloid fibroblast culture resulted in a dose-dependent decrease in the expression of collagen type I and fibronectin 72. In brief, the IL-6 signaling pathway has been implicated in both hypertrophic scars and keloid pathogenesis.

Systemic sclerosis (scleroderma, SSc) is an autoimmune connective tissue disease characterized by the production of autoantibodies, alterations in the vasculature and increased deposition of ECM in the skin and internal organs. Numerous studies have shown a high level of IL-6 in the serum of patients with SSc 73, and IL-6 expression is highly upregulated in SSc skin fibroblasts 74. In a hypochlorous acid-induced mouse model of systemic sclerosis, the mRNA expression of IL-6 was upregulated in skin and lung tissues 75. Trans-signaling of IL-6 through the JAK2/STAT3 and ERK pathways has been shown to have a significant profibrotic effect in vitro 76. As a result, IL-6 could be a therapeutic target for patients with SSc. A phase 2 trial of tocilizumab by Khanna et al. 77 showed preliminary evidence of the efficacy of this strategy in SSc. Recently, a phase 3 trial further assessed the safety and efficacy of tocilizumab in treating skin fibrosis and systemic sclerosis-associated interstitial lung disease (SSc-ILD). Although the primary skin fibrosis endpoint was not met, the secondary endpoint of the percentage of predicted forced vital capacity indicated that tocilizumab might preserve lung function in people with early SSc-ILD. Overall, these findings suggest that tocilizumab has a positive risk/benefit profile in SSc, warranting further investigation.

### IL-6 and ocular fibrosis

IL-6 has been implicated in numerous fibrotic eye diseases, including glaucoma filtration surgery (GFS), posterior capsular opacification (PCO) and proliferative vitreoretinopathy (PVR). We summarize several recent developments in the role and mechanism of IL-6 in fibrotic eye diseases as follows.

Glaucoma is a chronic progressive optic neuropathy that is sensitive to elevated intraocular pressure (IOP) and can lead to permanent loss of peripheral or central vision 78. Currently, the only available evidence-based treatment is to reduce IOP with topical drugs, lasers and surgery 79. GFS, which involves draining the aqueous humor into the filtration bleb in the conjunctiva, is widely used to reduce IOP. However, fibrosis and scarring in the subconjunctival space leading to the recurrence of elevated IOP are the major causes of surgical failure 80. RNA sequencing showed that the IL-6 gene was significantly upregulated in fibrotic fibroblasts isolated from patients with previous glaucoma surgery 81. Furthermore, compared to eyes with surgical failure, eyes with filtration surgical success, which is defined as an IOP less than 21 mm Hg without antiglaucoma medication, have significantly lower levels of IL-6, suggesting that following GFS, increased levels of IL-6 in the aqueous humor through the filtering passage into the sub-Tenon's space may enhance postoperative inflammation and contribute to fibrosis 82. Watanabe-Kitamura et al. 83 found that the expression levels of genes encoding IL-6 at the surgical site were elevated at 3 h following GFS, whereas the levels of fibrotic genes such as TGF-β and α-SMA were elevated at 3 days. In addition, TGF-β-induced α-SMA expression was inhibited by IL-6 trans-signaling, suggesting that IL-6 can delay the phase transition from inflammation to proliferation. The findings in the present study suggest that IL-6 is important for scar wound repair in GFS. However, further research is needed to understand the mechanism of IL-6 in GFS fibrosis.

PCO, which is the most common complication of cataract surgery, is a consequence of surgical injury initiating a wound healing response that ultimately results in a reduction in visual quality 84. PCO occurs when residual lens epithelial cells undergo fibrotic changes, such as hyperproliferation, migration, ECM deposition and epithelial cell transdifferentiation to a myofibroblast phenotype 84. PCO is more severe and frequent in young patients than in elderly patients. Human capsular bags of young and elderly individuals were cultured in serum-free Eagle's minimum essential medium, and a significant increase in IL-6 was observed in young counterparts 85. TGF-β2, which is an important factor in the development of PCO, can be upregulated in the presence of IL-6 via a JAK/STAT3 signaling-dependent mechanism 86. Moreover, inhibiting JAK/STAT3 signaling effectively prevented PCO development in vitro and in vivo.

PVR, which is a scarring process characterized by the formation of fibrous membranes on the epiretinal surface, is a severe complication of ocular trauma, retinal detachment and inflammatory vitreoretinopathies. It was found that the concentration levels of IL-6 are significantly upregulated in the vitreous of PVR patients 87. Consistently, Symeonidis et al. 88 demonstrated that IL-6 levels were increased in the subretinal fluid and were positively correlated with PVR grade. PVR membranes are composed of a variety of cell types, including fibroblasts, macrophages, and retinal pigment epithelial (RPE) cells. Of these cell types, RPE cells account for the largest proportion and are considered to play a crucial role in the pathogenesis of PVR by transitioning from the epithelial to mesenchymal phenotype. It was reported that treating RPE cells with IL-6 promoted cell proliferation, induced morphological changes from epithelial to fibroblast-like cells, and upregulated mesenchymal markers by activating the JAK1/STAT3 signaling pathway in vitro 89. In addition, IL-6 knockout in a PVR mouse model markedly prevented PVR progression 89. The present evidence suggests that blocking IL-6 may be a promising strategy for the prevention and treatment of PVR.

## Conclusions and the future

In this review, we gathered evidence about the role of IL-6 in fibrosis and recent research on IL-6 in different fibrotic tissues and organs. According to previous research results, we can conclude that IL-6, which is a pleiotropic cytokine that plays an important role in inflammation, immunity and hematopoiesis, is a critical cytokine that drives the development of fibrosis. Given its possible role in the pathophysiology of fibrosis, IL-6 has been suggested to be a useful fibrotic biomarker. Previous studies on fibrosis have mostly focused on TGF-β and its downstream signaling pathway; however, the safety and efficacy of drugs targeting TGF-β for the treatment of fibrosis are not yet clear. Although nintedanib and pirfenidone have been approved by the Food and Drug Administration (FDA) for fibrotic disease treatment, these drugs only delay disease progression 90. Therefore, it is very important to find new antifibrotic targets, and inhibitors of IL-6 and its signaling pathway may be new agents for resisting fibrosis. Understanding the signaling pathways and role of IL-6 in fibrosis provides an opportunity to develop therapies to attenuate the development of fibrotic diseases and facilitate the resolution of fibrotic injury.

The FDA has approved agents that target IL-6 and downstream signaling pathways, such as IL-6, IL-6R, and JAKs, for the treatment of inflammatory diseases and myeloproliferative neoplasms 10. Novel inhibitors of the IL-6/JAK/STAT3 pathway are also being developed, including STAT3-selective medicines, and early phase clinical trials are now underway. Many of these drugs are also being investigated as potential treatments for other pathological conditions. In addition, immune-mediated adverse effects are one concern of IL-6 treatments. As we discussed above, proinflammatory IL-6 trans-signaling plays an important role in the development of fibrosis. Therefore, in future drug research and development, to reduce side effects, targeting the trans-signaling pathway of IL-6 to alleviate fibrosis should be a focus of attention.

In conclusion, inhibiting the IL-6/JAK/STAT3 signaling axis, which has previously been shown to be effective in the treatment of fibrosis, holds considerable promise for inhibiting fibrotic development. In an attempt to optimize the general application and efficacy of medicines targeting the IL-6/JAK/STAT3 signaling pathway and their use in precision medicine for patients with fibrosis, predictive biomarkers and rational combination therapy based on the immunological and genetic profiles of fibrosis are needed.

## Abbreviations

ECM: extracellular matrix
IL-6: interleukin 6
JAK: Janus kinase
STAT3: signal transducer and activator of transcription 3
α-SMA: alpha-smooth muscle actin
IL-6R: IL-6 receptor
gp130: glycoprotein 130
mIL-6R: membrane-bound IL-6R
sIL-6R: soluble IL-6R
MAPK: mitogen-activated protein kinase
SOCS: suppressor of cytokine signaling
Grb2-SOS: growth-factor-receptor-bound protein 2/Son of Sevenless
MCP-1: monocyte chemoattractant protein 1
TGF-β: transforming growth factor β
FMT: fibroblast-to-mesenchymal transition
CaMKII: Ca^2+^/calmodulin-dependent protein kinase I
EMT: epithelial-mesenchymal transition
NSCLC: non-small cell lung cancer
IPF: idiopathic pulmonary fibrosis
HMVECs: human microvascular endothelial cells
atg5: autophagy-related 5
COVID-19: coronavirus disease 2019
CKD: chronic kidney disease
SSc: systemic sclerosis
SSc-ILD: systemic sclerosis-associated interstitial lung disease
GFS: filtration surgery
PCO: posterior capsular opacification
PVR: proliferative vitreoretinopathy
IOP: intraocular pressure
RPE: retinal pigment epithelial
FDA: Food and Drug Administration
